# Screening of Lynch syndrome in endometrial cancer in Iranian population with mismatch repair protein by immunohistochemistry

**DOI:** 10.22088/cjim.13.4.772

**Published:** 2022

**Authors:** Somayyeh Noei Teymoordash, Maliheh Arab, Massih Bahar, Abdolali Ebrahimi, Maryam Sadat Hosseini, Farah Farzaneh, Tahereh Ashrafganjoei

**Affiliations:** 1 Department of Obstetrics and Gynecology, Firoozgar Hospital, Iran University of Medical Sciences (IUMS), Tehran, Iran; 2 Department of Gynecology Oncology, Imam Hossein Medical Center, Shahid Beheshti University of Medical Sciences, Tehran, Iran; 3 Familial and Hereditary Cancers Institute, Tehran, Iran; 4 Department of Pathology, Imam Hossein Medical Center, Shahid Beheshti University of Medical Sciences, Tehran, Iran; 5 Preventative Gynecology Research Center (PGRC), Imam Hossein Medical Center, Shahid Beheshti University of Medical Sciences, Tehran, Iran

**Keywords:** Endometrial cancer, Microsatellite instability, Mismatch repair protein deficiency, Lynch

## Abstract

**Background:**

Lynch syndrome (LS) is one of the commonest genetic cancer syndromes, with an incidence rate of 1 per 250–1000 population. The aim of this study was to evaluate the frequency and characteristics of MMR deficiency in endometrial cancer in Iranian women.

**Methods:**

One hundred endometrial carcinoma cases who referred to the gynecological oncology clinic of Imam Hossein Medical Center located in Tehran, Iran, from 2018 to 2020 were included in the study. Immunohistochemistry (IHC) evaluation was performed mainly on the hysterectomy specimens of all endometrial cancer (EC) patients to assess MMR proteins (MLH1, MSH2, MSH6, and PMS2) expression.

**Results:**

A total of 23 out of 100 (23%) cases were identified through IHC screening to be MMR-deficient. The most common types were loss of MLH1/PMS2 (17.4%) and solitary MSH2 (17.4%) expressions followed by PMS2/MSH2 loss (13%). MMR deficiency (dMMR) histopathology was significantly overrepresented in patients with family history of cancer or Lynch syndrome (LS) associated cancers (p-values of 0.016 and 0.005, respectively). The rate of myometrial invasion and lower uterine segment involvement were also significantly higher in dMMR EC patients compared to MMR-intact EC (p-value of 0.021 and 0.018, respectively).

**Conclusion:**

MMR deficiency, observed in 23% of endometrial cancer cases, was associated with higher rates of poor prognostic factors including myometrial invasion and lower uterine segment involvement. The presence of positive family history of cancer and family history of LS-associated cancer increased the probability of MMR-deficiency in endometrioid endometrial cancer to 47% and 70%, respectively.

Lynch syndrome (LS) is one of the commonest genetic cancer syndromes, with an incidence rate of 1 per 250–1000 population (1). Genetic defect in one of the four mismatch repair genes i.e., MSH2, MLH1, MSH6, PMS2, or in the EPCAM gene, is the etiology behind this autosomal dominant disease (2, 3). MMR deficiency (dMMR) from loss of MMR functioning, causes microsatellite instability (MSI), hypermutated phenotype, and increased cancer susceptibility (4). Development of LS in colorectal cancer (CRC), endometrial cancer (EC), and various other LS-associated cancers (LS-AC), occurs at early ages compared to the general population. Women with pathogenic germline MMR gene mutation have 43-48% lifetime risk of developing CRC; the risk is 40%–62% for EC, 2%–13% for gastric cancer (GC), and 6%–14% for ovarian cancer (OC), while the risk of developing other LS-AC also increases greatly. LS accounts for an estimated 2%–6% of EC patients.

The recommendation from Society for Gynecology Oncology (SGO) is to identify high risk patients through a systematic clinical screening for Lynch syndrome in all endometrial cancer patients by reviewing their personal and family history, followed by germline or molecular tumor testing of high-risk patients. However, SGO considers tumor testing on all endometrial cancers or on those diagnosed before 60 years of age, to be a more sensitive approach than the abovementioned clinical screening. Many recently published international guidelines, including those from the American College of Obstetricians and Gynecologists (ACOG), the Society of Gynecologic Oncology (SGO) and the National Comprehensive Cancer Network (NCCN), have considered the performance of the universal tissue testing in all newly diagnosed EC cases to be a valuable approach (5). 

Identification of LS is typical through cancer patients, and tumor-based triage tests (immunohistochemistry [IHC] for MMR protein loss, MSI testing or MLH1 promoter methylation testing) are the means which identify the cases needing to undergo germline testing for identification of the MMR gene pathogenic variant (3, 4). Pathogenic germline variants of Lynch syndrome genes (MLH1, MSH2, MSH6 and PMS2) are responsible for nearly 13%-25% of MMR-deficient ECs, while in 62%–73% of cases, somatic hypermethylation of the promoter region of the MLH1 gene is the culprit (5). 

MMR-IHC is one of the two screening modalities (the other being the molecular MSI analysis) which analyzes the expression of MLH1, MSH2, MSH6 and PMS2 proteins. It is an efficient, easy to perform, widely available test with moderate cost; advantages that have enabled most modern pathology laboratories and pathologists to have the expertise for appropriate analysis and result interpretation. For patients exhibiting IHC loss of MLH1, a PCR-based MLH1 promoter methylation assay is performed to distinguish the cases of somatic hypermethylation from potential LS (6). MMR status determination in all endometrial cancer patients is recommended, firstly as a tool to diagnose LS, and secondly for its predictive, prognostic, and therapeutic values (1). The few related studies in literature have reported conflicting results, given their heterogeneity. Some have correlated MMR deficient status with poor outcomes, while others have reported a better prognosis and response to adjuvant therapies in this group. The present study reports the experience of universal IHC screening for MLH1, PMS2, MSH2, and MSH6 deficiencies in EC patients from 2018 to 2020.

## Methods

All endometrial carcinoma cases referred to the gynecological oncology clinic of Imam Hossein Medical Center from 2018 to 2020 were included in this study. Lack of access to information, and patient’s unwillingness to participate in the study were the exclusion criteria. After obtaining relevant informed consent, detailed epidemiological, clinical, and pathological data were retrieved from the electronic medical record, including: age at diagnosis, body mass index (BMI), parity, menstrual status, comorbidities, tumor size, FIGO surgical stage, tumor histology, FIGO tumor grade (non-endometrioid tumors were considered as grade 3), depth of myometrial invasion, lower uterine segment involvement, presence of lympho-vascular invasion (LVSI), involvement of uterine cervix, serosa, ovaries, and lymph nodes.

**Screening protocols: **To assess MMR protein (MLH1, MSH2, MSH6, and PMS2) expression, IHC was performed on the blocks and slides of hysterectomy specimens from all EC patients, according to standard procedures. In cases where the patient was not candidate for surgery due to a medical problem or fertility preservation and in cases with inadequate hysterectomy specimens (no tumor residue), MMR IHC staining was performed on samples of endometrial biopsy specimens. The specimen was examined by a pathologist experienced in gynecological oncology for four MMR proteins including MLH1, MSH2, MSH6, and PMS2. An appropriate paraffin-embedded tissue was cut with 2-4-μm thickness. The tissue sections were deparaffinized in xylene and were rehydrated in graded alcohol. Then, antigen retrieval was done in a microwave oven for 20 minutes. These sections were allowed to cool at room temperature. Next, the primary antibodies were used overnight at 4 °C. After hematoxylin staining, adjacent normal endometrium and lymphocytes in the slides were used as positive internal controls. The complete loss of nuclear staining in the tumor cells was considered as MMR deficiency. The result of IHC MMR was interpreted by the pathologist as intact (normal) and deficient (abnormal). Deficient results were reported in the absence of any of the four MMR proteins in the IHC sample. Patients with IHC loss of MSH2, MSH6, PMS2 or MLH1 with lack of methylation were referred to a genetic counselor for germline testing. In this study, the subjects’ information remained confidential; no change was made in their diagnostic and treatment processes and no cost was imposed on them. The study protocol was verified by the Ethics Committee of Shahid Beheshti University of Medical Sciences (IR.SBMU.RETECH.REC.1399.944). All cases signed the informed consent form.

**Statistical analyses: **The data were collected using checklist and were analyzed using SPSS23 statistical software. Quantitative data were expressed as mean±sd (standard deviation), while qualitative data were represented by frequency and percentage. Chi-square test and independent t-test were used to compare clinical and pathological features between MMR-deficient EC and MMR-intact EC and univariate logistic regression was used to examine the relationship between each variable and the MMR state. A p-value of <0.05 was considered significant.

## Results

Patients’ demographic, clinical, and tumor-related characteristics are summarized in table 1. A total of 23 out of 100 cases were identified through IHC screening to be MMR-deficient. Most frequent MMR-deficiency states were related to the loss of expression of MLH1/PMS2 (17.4%) and solitary MSH2 (17.4%) followed by PMS2/MSH2 loss (13%). Comparison of demographic and clinical features between suspected Lynch syndrome (MMR-deficient) and MMR-intact endometrial cancer patients is demonstrated in table 2. There was a statistically significant difference in presence of the family history of cancers and the family history of Lynch-associated cancers between the two groups. Positive family history of malignancy in the MMR-intact EC was observed in 11 (14.3%) patients; these included breast cancer (3 cases), lung cancer (3 cases), gastric cancer (2 cases), hepatobiliary cancer (2 cases), and brain tumor (1 case). In the MMR-deficient EC group, 9 (39.1%) patients had positive family history of malignancy, including colorectal cancer (4 cases), gastric cancer (2 cases), breast cancer (2 cases), and endometrial cancer (1 case).

Comparison of pathological features between suspected Lynch syndrome and MMR-intact endometrial cancer are shown in table 3. The rate of myometrial invasion and lower uterine segment involvement was significantly higher in the former group. Incidence of variables such as cervical involvement, LVSI, and need for adjuvant treatment was also noted to be higher in the MMR-deficient EC group, although these differences were statistically non-significant.

**Table 1:** Clinicopathological characteristics of the studied population

Age Mean ± SD (Range)		56.59±10.85 (31-85)
BMI* Mean±SD (Range)		31.27±6.39 (17.7-52)
Tumor size Mean±SD (Range)		35.08±23.13 (0-140)
Myometrial invasion (%) Mean±SD (Range)		42.80±31.65
Histology N (%)	Endometrioid	91 (91%)
	Non-Endometrioid	9 (9%)
Grade N (%)	I	49 (49%)
	II	29 (29%)
	II	22 (22%)
Stage N (%)	Early (I,II)	85 (84.2%)
	Late (III, IV)	25 (15.8%)

*BMI: Body mass Index

**Table 2:** Comparison of clinical characteristics between MMR-deficient and MMR-intact

**Variable**	**Intact** **(n=77)**	**Deficient** **(n=23)**	**P-value**	**OR (95% CI)**
Age>60	30 (39)	6 (26.1)	0.259	0.533 (0.196-1.56)
‌BMI>30	46 (59.7)	11 (47.8)	0.311	0.618 (0.242-1.58)
Parity				
Nulligravid	15 (19.5)	5 (21.7)	0.953	
Primiparous	8 (10.4)	2 (8.7)		0.750 (0.118-4.77)
multiparous	54 (70.1)	16 (69.6)		0.889 (0.280-2.82)
Personal history of LS-associated cancer	0 (0)	1 (4.3)	0.230	
Previous history of cancer	6 (7.8)	1 (4.3)	0.999	0.538 (0.061-4.71)
Hypertension	39 (50.6)	13 (56.5)	0.621	1.27 (0.496-3.24)
Diabetes Mellitus	21 (27.3)	6 (26.1)	0.911	0.941 (0.321-2.71)
Hyperlipidemia	12 (15.6)	3 (13)	0.999	0.738 (0.191-2.85)
Hypothyroidism	13 (16.9)	2 (8.7)	0.510	0.469 (0.098-2.25)
Cardiac disease	17 (22.1)	6 (26.1)	0.688	1.25 (0.425-3.65)
Family history of LS-associated cancer	5 (6.5)	7 (30.4)	0.005	6.30 (1.77-22.41)
Family history of cancer	11 (14.3)	9 (39.1)	0.016	3.86 (1.35-11.06)

**Table 3:** Comparison of pathological characteristics between MMR-deficient and MMR-intact

**Variable**	**Intact** **(n=77)**	**Deficient** **(n=23)**	**P-value**	**OR (95% CI)**
Size tumor (mean± SD)	36.24±21.33	31.17±28.56	0.359	
Endometrioid histology	69 (89.6%)	22 (95.6%)	0.374	0.392 (0.46-3.31)
Lower segment	20 (26)	12 (52.2)	0.018	3.11 (1.19-8.16)
Myometrium invasion	39.42 ± 33.11	54.13 ± 23.44	0.021	1.02 (1.01-1.03)
Cervical involvement	10 (13)	6 (26.1)	0.191	2.37 (0.754-7.42)
Ovarian involvement	11 (14.3)	2 (8.7)	0.727	0.571 (0.117-2.79)
Serosal involvement	3 (3.9)	0 (0)	0.999	
Positive cytology	1 (1.3)	1 (4.3)	0.999	3.46 (0.208-54.50)
Lymph node involvement	11 (14.3)	2 (8.7)	0.727	0.571 (0.117-2.79)
LVSI*	17 (22.1)	8 (34.8)	0.217	1.88 (0.684-5.18)
Omental involvement	2 (2.6)	1 (4.3)	0.548	1.71 (0.148-19.70)
Stage:			0.335	0.469 (0.98-2.25)
Early (I+II)	64 (83.1)	21 (91.30)		
Late (III+IV)	13 (16.9)	2 (8.70)		
Adjuvant therapy	44 (57.1)	17 (73.9)	0.148	2.13 (0.755-5.98)
Brachytherapy	38 (49.4)	14 (60.9)	0.332	1.60 (0.618-4.12)
EBRT	24 (31.2)	7 (30.4)	0.947	0.966 (0.352-2.65)
Chemotherapy	24 (31.6)	6 (26.1)	0.616	0.765 (0.268-2.18)

*LVSI: Lympho-vascular space invasion

**Table 4:** Prevalence of Suspected LS and LS in different studies

**Authors, year**	**Population** **(N)**	**MMR-deficient (suspected LS)** **N (%)**	**MMR-deficient (suspected LS) in EEC** ^˨^ **N (%)**	**Most prevalent** **MMR-deficient**	**LS in** **MMR-deficient** **N (%)**	**LS in EC** ^˨^ **(%)**	**LS in EEC** **N (%)**
Arab M et al, 2021	100	23 (23)	22 (24.17)				
Ismael, 2020 (17)	60	28 (45)		All loss, PMS2			
Dondi, 2020 (5)	239	96 (40)		MLH1, PMS2	18 (18.75)	7.5	
Gordhandas, 2020 (18)	7057	1612 (23)		MLH1	212/900 (24)	3	
Saeki, 2019 (19)	98	23 (23.5)					
Reijnen*, 2019 (7)	128	57 (44.5)					
Chao, 2019 (20)	111	26 (23.5)		MLH1/PMS2	6 (23%)	5.4	6/87(6.89)
Kahn, 2019 (21)	5917	1672 (28)		MSH2	206(12.3%)	3	
Backes*, 2019 (8)	197	64 (32.48)	64 (32.48)				
Kim, 2018 (22)	173˨	45 (26)	45 (26)	MLH1/PMS2			
Cosgrove, 2017 (23)	466	116 (24.9)		MLH1/PMS2			
Mass-Moya. 2016 (16)	215	72 (33)			11/52 (21.15)		
Mills, 2016 (10)	210	66 (31.4)		MLH1, PMS2	7/55 (26.2)	3.33	
Buchanan, 2014 (24)	702	170 (24)		MLH1/PMS2	22 (13)	3	21/572 (3.7)
Ferguson, 2014 (25)	118	34 (29)		MLH1, PMS2	7 (20.58)	5.9	
Peterson, 2012 (26)	98	23 (24)		MLH1/PMS2			
Total	15087	4104 (27.20)	131/461 (28.4)		489/3005 (16.27)	489/14354 (3.40)	27/659 (4.11)

EC: Endometrial cancer, EEC: Endometrioid endometrial cancer *In Backes and Reijnen studies, high risk population were included

**Table 5:** Frequency of MMR-deficiency in endometrioid endometrial cancer in various status

Possibility of LS* **(%)**	**MMR-deficient** **N (%)**	**Clinical scenario**
7.65	8/17 (47)	FH of cancer (+)
3.1	14/74 (19)	FH of cancer (-)
11.4	7/10 (70)	FH of LS-associated cancer (+)
3	15/81 (18.5)	FH of LS-associated cancer (-)
3.94	22/91 (24.2)	Total

*LS: Lynch Syndrome

**Table 6:** Comparison of some pathological features with MMR status in different studies

**Author**	**High grade** **(%)**		MI*≥ 50%		LVSI* **(%)**		LUS* **(%)**		**Early stage** **(%)**		**Adjuvant therapy**	
	**iMMR**	**dMMR**	**iMMR**	**dMMR**	**iMMR**	**dMMR**	**iMMR**	**dMMR**	**iMMR**	**dMMR**	**iMMR**	**dMMR**
Arab et al.,2021	23.37	21.05	39.42 mm	54.13 mm	22.1	34.8	26	52.2	83.1	91.30	57.1	73.9
	P: .543		P: .021		P: .217		P .018		P .018		P: .148	
Gordha et al., 2020	20	29							88	80		
	p<0.01								p<0.01			
Backes et al, 2019	9	28.1	70.7	57.8	33.1	45.3	9.4	27.8			45.9	43.8
	p<0001		p: .073		p: .096		p<0001				p .879	
Kim KS et al. 2018	58.2	50			53.4	65.4			79	71.6		
	P: .003				p: .007				p: .13			
Nagle et al, 2018	21.9	25			23	33.74			88.3	86.9	34	45.4
	p: .028				p: .02				p: .44		p: .03	
Kim J et al, 2018	42.5	64.5	28.6	40	12.1	31.8			91.4	75.6	23.4	44.4
	p: .011		p: 0.15		p: .003				p: .014		p: .007	
Cosgrove et al, 2017	17.1	21.55	26.9	34.5	18.5	40.5			83.7	71.5	29	40.9
	p: .002		p: .024`		p: <.001				p: .015		p: .045	
McMeekin et al, 2016	13.14	18.45	25.68	28.98	17.25	32.23			86.7	80.44		
	p: .01		NS		p: .001				p: .001			

MI: Myometrial invasion; LVSI: Lympho-vascular space invasion; LUS: lower uterine segment

## Discussion

In the present study, among 100 cases of endometrial cancer, the frequency of MMR-deficient cases was 23 (23%) and the most common MMR-deficient cases were MLH1 / PMS2-deficient (4/23; 17.39%) and solitary MSH2-deficient (4/23; 17.39%). Other studies also reported MLH1 / PMS2 deficiency to be the most common MMR deficiency (table 4). Different studies have reported the frequency of MMR-deficiency to be 23% to 25%, though higher rates of MMR-deficiency have been reported by Reijnen et al. (7) (44.5%) and Backes et al. (8) (32.48%), possibly due to the high-risk population selected in these studies. Studies have reported the prevalence of Lynch syndrome to be 12% to 24% among suspected LS (MMR-deficient) cases. Among those with endometrial cancer, 3% to 7.5% are reported to have Lynch syndrome; this rate is found to be 3.7% to 6.89% in endometrioid endometrial cancer (table 4). For better understanding, pooling the cases presented in different studies revealed the mean prevalence of Lynch syndrome is 16.27% (489/3005) in suspected LS (MMR-deficient) cases and 3.40% in endometrial cancer patients (table 4). Therefore, with the prevalence of 23% for MMR-deficiency in the present study, incidence of Lynch syndrome is estimated to be around 3.70%. Hence, the possible prevalence of Lynch syndrome in the study population is comparable to those reported in different studies.

In the present study, the family history of cancer and the family history of Lynch-associated cancer were 39.1% and 30.4% in the MMR-deficient (suspected LS) group respectively, which was significantly different compared to the MMR-intact group (table 2). In the study by Takahashi et al., personal history of cancer and family history of Lynch-associated cancer were 50% and 100% in LS, respectively (9). In the study by Mills et al., family history of Lynch-associated cancer was observed in 28.5% (2/7) of LS patients and 12.5% (1/8) of Lynch-like (LL) patients (10), so it can be concluded that the family history of cancer, especially that of lynch-associated cancer, is strongly associated with MMR-deficiency. In Iran, genetic testing is not provided by public health sector. To assess the long-term benefits of testing in different subgroups, frequency of positive test for MMR deficiency is calculated in these subgroups, as depicted in table 5. In the present study, the probability of MMR-deficiency in endometrioid endometrial cancer (EEC) in the presence of positive family history of cancer increases to 47% and in cases of a positive family history of Lynch-associated cancer, to 70%. Therefore, germline testing in EEC cases with a positive family history of cancer, especially of lynch-associated cancers is more cost-effective in limited resource settings (table 5).

In the present study, the rates of deep myometrial invasion and lower segment involvement, which are factors with unfavorable prognosis, were significantly higher in MMR-deficient patients compared to the MMR-intact group (table 3). Some studies have evaluated the histological characteristics of MMR-deficient endometrial tumors. These studies have revealed most of the MMR-deficient tumors to be of endometrioid type. It is noteworthy that compared to non-endometrioid tumors, endometrioid histology has a better prognosis. The relationship between MMR deficiency and outcomes in EC patients is not well understood; different studies have provided conflicting results, some reporting better survival rates in women with MMR deficient EC, while others reporting this population to have poorer outcomes with higher rates of poor prognostic factors (e.g., advanced stage disease, deep myometrial invasion and LVSI). Comparison of some of the pathological and therapeutic features between the MMR-deficient and MMR-intact endometrial cancer in different studies is shown in table 6. As noted in table 6, in majority of the studies, features such as high-grade tumors, deep myometrial invasion, LVSI, advanced stage (III, IV), and the need for adjuvant treatment were more common in the dMMR group. Backes et al. (2019) found 5-year recurrence-free survival (RFS) rates of 66% in dMMR patients and 89% in iMMR cases (p: .001) (8). In a meta-analysis conducted by Xiao Jingping in 2020 (assessing 7 studies and 1150 patients with early-stage EEC), progression free survival (PFS), disease free survival (DFS), and overall survival (OS) were found to be lower in dMMR patients compared to the iMMR cases (P: .006) (11). In Fountzila’s study, MMR-deficiency was associated with improved OS in EC patients (HR = 0.38, 95% CI= 0.20 - 0.76, P=0.006) (12). While interpreting studies that report controversy in recurrence or survival in dMMR cases, few points are worth considering. Firstly, dMMR is more prevalent among endometrioid type cancer, which in itself is associated with a better prognosis compared to non-endometrioid histology. Secondly, Kim KS and Reijnen's studies revealed a higher sensitivity to radiotherapy in dMMR cases; this also could result in improved prognosis in MMR-deficient patients. Thirdly, the patient selection was different in various studies. Studies that included only endometrioid type cancers, such as Kim and Backes studies, mostly showed a worse prognosis in dMMR cases. Hence MMR protein status determination can aid in predicting prognosis and possible response to adjuvant therapy.

MMR-protein status also has a role in treatment planning. For example, in MMR-deficient women under 55 years of age who suffer from atypical complex hyperplasia or well-differentiated EC, progesterone treatment is ineffective (13). On the other hand, selective pathway blockade with PD-1 inhibitors is highly considered in MMR-deficient tumors; this is due to upregulation of immune checkpoints such as the programmed death 1 (PD-1) pathway, by such tumors. PD-L1 expression occurs in 52.6% of dMMR-ECs, but only in 10% of MMR-intact cases (14). In May 2017, pembrolizumab (an immune checkpoint inhibitor) was approved by the US Food and Drug Administration (FDA) for treatment of non-resectable solid tumors with MMR-deficiency or high MSI, irrespective of tumor location (15). Therefore, the MMR-protein status can influence the treatment choice, especially in the setting of disease progression or recurrence. It should be noted that such treatment can be considered in suspected-LS cases, even without knowing the germline status.

A major challenge in the universal LS screening in EC patients is the identification of MMR-deficient patients without any pathogenic germline mutation (Lynch-like (LL) syndrome). It is not clear whether patients with LL syndrome should use the same lifelong invasive screening protocol that has been approved for patients with LS. In a study conducted by Mas-Moya, there was no significant difference in clinicopathological features between LL-associated EC and Lynch syndrome-associated EC (16). Loss of MSH2 or MSH6 expression in IHC often indicates definite Lynch syndrome. Some centers offer recommendations regarding colorectal cancer screening, and gynecological risk-reducing surgeries for these patients and their family members, as well as for patients with definite Lynch syndrome. The present study has limitations such as the relatively small sample size as well as the limited follow-up time to compare overall survival between the two groups. It is nonetheless the first single-institution experience with universal MMR IHC testing in Iranian EC patients. MMR deficiency was observed in 23% of endometrial cancer cases in a public medical center in Tehran, Iran. 

The probability of MMR-deficiency in endometrioid endometrial cancer (EEC) in the presence of a positive family history of cancer increases to 47%, and a positive family history of Lynch-associated cancer raises the risk to 70%. MMR deficiency was associated with higher rates of occurrence of some of the poor prognostic factors, including myometrial invasion and lower uterine segment involvement.
